# Maternal Concentrations of Polyfluoroalkyl Compounds during Pregnancy and Fetal and Postnatal Growth in British Girls

**DOI:** 10.1289/ehp.1003096

**Published:** 2012-08-30

**Authors:** Mildred Maisonet, Metrecia L. Terrell, Michael A. McGeehin, Krista Yorita Christensen, Adrianne Holmes, Antonia M. Calafat, Michele Marcus

**Affiliations:** 1 Department of Epidemiology, Rollins School of Public Health, Emory University, Atlanta, Georgia, USA; 2 National Center for Environmental Health, Centers for Disease Control and Prevention, Atlanta, Georgia, USA; 3 Kaiser Permanente Center for Health Research, Atlanta, Georgia, USA

**Keywords:** ALSPAC, birth weight, early childhood growth, perfluorohexane sulfonate, perfluorooctanoate, perfluorooctane sulfonate, polyfluoroalkyl compounds, postnatal growth

## Abstract

Background: Prenatal exposures to polyfluoroalkyl compounds (PFCs) may be associated with adverse changes in fetal and postnatal growth.

Objective: We explored associations of prenatal serum concentrations of perfluorooctane sulfonate (PFOS), perfluorooctanoate (PFOA), and perfluorohexane sulfonate (PFHxS) with fetal and postnatal growth in girls.

Methods: We studied a sample of 447 singleton girls and their mothers participating in the Avon Longitudinal Study of Parents and Children (ALSPAC). Data on weight and length were obtained at birth and at 2, 9, and 20 months. Serum samples were obtained in 1991–1992, from mothers during pregnancy. We explored associations between prenatal PFC concentrations and weight at birth as well as longitudinal changes in weight-for-age SD scores between birth and 20 months.

Results: PFOS (median, 19.6 ng/mL), PFOA (median, 3.7 ng/mL), and PFHxS (median, 1.6 ng/mL) were detected in 100% of samples. On average, girls born to mothers with prenatal concentrations of PFOS in the upper tertile weighed 140 g less [95% confidence interval (CI): –238, –42] at birth than girls born to mothers with concentrations in the lower tertile in adjusted models. Similar patterns were seen for PFOA (–133 g; 95% CI: –237, –30) and PFHxS (–108 g; 95% CI: –206, –10). At 20 months, however, girls born to mothers with prenatal concentrations of PFOS in the upper tertile weighed 580 g more (95% CI: 301, 858) when compared with those in the lower tertile. No differences in weight were found for PFOA and PFHxS.

Conclusions: Girls with higher prenatal exposure to each of the PFCs examined were smaller at birth than those with lower exposure. In addition, those with higher exposure to PFOS were larger at 20 months.

Polyfluoroalkyl compounds (PFCs) are used in the production of fluoropolymers, which have applications in protective coatings of packaging products, clothes, furniture, and nonstick cookware. Some PFCs are persistent compounds, ubiquitous in the environment, and human exposure is common (Agency for Toxic Substances and Disease Registry 2009). PFCs have been detected in human sera, breast milk, and cord blood (Apelberg et al. 2007; Calafat et al. 2007; Karrman et al. 2007).

Exposure to perfluorooctane sulfonate (PFOS), perfluorooctanoate (PFOA), and perfluorohexane sulfonate (PFHxS)—three of the most studied PFCs—may have deleterious effects on fetal growth. Lower body weights at birth were reported in the offspring of rats exposed to PFOS and of mice exposed to PFOA during pregnancy (Hines et al. 2009; Lau et al. 2003, 2006; Luebker et al. 2005; White et al. 2007; Wolf et al. 2007). In humans, maternal serum concentrations of PFOS during pregnancy have been inversely associated with birth weight (Washino et al. 2009) and with higher risk of low birth weight (Stein et al. 2009), and maternal serum concentrations of PFOA have been inversely associated with birth weight (Stein et al. 2009) and length (Apelberg et al. 2007). Cord serum concentrations of both PFOS and PFOA were not associated with birth weight or length but were inversely associated with head circumference and ponderal index (Apelberg et al. 2007). However, other studies did not find associations between maternal serum concentrations of PFOS, PFOA, or PFHxS and fetal growth in their offspring (Hamm et al. 2010; Nolan et al. 2009).

One of the proposed mechanisms by which PFCs may affect growth is through activation of the peroxisome proliferator-activated receptor alpha (*PPAR*α) (Abbott et al. 2007). The peroxisome proliferator-activated receptor (PPAR) family is composed of three isotypes (PPARα, β, and γ), which play an important role in control of cellular differentiation programs and in the transcriptional control of lipids and carbohydrate metabolism (Casals-Casas et al. 2008). Mice prenatally exposed to PFOA who express the *PPAR*α gene had lower postnatal body weights than exposed knockout mice that do not express the *PPAR*α gene (Abbott et al. 2007). In mice prenatally exposed to PFOS, however, postnatal body weight was not dependent on *PPAR*α expression (Abbott et al. 2009). In a separate study, higher body weights in midlife were reported in female offspring of mice exposed to PFOA prenatally (Hines et al. 2009).

Altered development of adipose tissue and of the regulatory systems involved in body weight homeostasis may explain inverse associations of PFOS and PFOA serum concentrations with measures of fetal growth reported in the epidemiologic literature. The influence of prenatal exposures to PFCs on weight homeostasis may persist after birth, although evidence is limited and somewhat inconsistent. The purpose of this investigation was to explore associations of maternal serum concentrations of PFOA, PFOS, and PFHxS during pregnancy with markers of fetal and postnatal growth in girls.

## Methods

*Population.* The Avon Longitudinal Study of Parents and Children (ALSPAC) enrolled pregnant women from three health districts of the county of Avon, Great Britain, with an expected delivery date between April 1991 and December 1992. A total of 14,610 children joined the cohort at birth. Details of recruitment methods are described elsewhere (Golding et al. 2001).

In 2004–2005, when enrolled offspring were 13 years old, there were 11,820 singleton active participants in the ALSPAC, of whom 5,756 (49%) were girls. Of these girls, 3,682 had returned at least two valid assessments of pubertal status between the ages of 8 and 13 years. We obtained a sample of 448 girls from the group of girls with at least two valid pubertal assessments for a study of maternal serum concentrations of PFCs and menarche (Christensen et al. 2011). Cases (*n* = 218) consisted of all the girls who attained menarche < 11.5 years, and controls (*n* = 230) were a random sample of noncases who attained menarche ≥ 11.5 years of age. We used this sample to study associations of maternal exposures to PFCs, estimated from the serum concentrations, with fetal and postnatal growth. Median age at menarche in the full cohort was 12.93 years (Rubin et al. 2009).

*Data collection.* Birth weight (in grams), length (in centimeters), and gestational age (in weeks) were abstracted from medical records. Weight and height at 2 (mean ± SD = 1.7 ± 0.3), 9 (9.2 ± 0.9), and 20 (19.7 ± 2.8) months were obtained by health professionals as part of the routine infant health surveillance program. We converted the infant’s weights to weight-for-age SD scores (*z*-scores) using the 1990 British growth reference curves for girls (Cole et al. 1995). Ponderal index was calculated using the following formula: (weight in grams/length in cubic centimeters) × 100. Self-reported data on maternal prenatal characteristics and behaviors were obtained from the mothers during pregnancy. Breast-feeding data were obtained from questionnaires administered when the girls were 4 weeks old. Data collection instruments and methods have been described in detail elsewhere (Golding et al. 2001). Potential confounders assessed include gestational age (weeks); maternal educational level (< O level/O level/> O level); maternal prepregnancy body mass index (BMI); maternal age at delivery (years); previous live births (0/≥ 1); maternal smoking during pregnancy (yes/no); mother’s ethnic background (white/nonwhite); breast-feeding of child between birth and 4 weeks (yes/no); and gestational age when maternal serum sample was obtained. An O-level education is the qualification obtained at age 16 years when obligatory schooling ends. We assigned maternal prepregnancy weight status according to the Centers for Disease Control and Prevention (CDC) adult BMI classification: underweight (< 18.5), normal (18.5–24.9), overweight (25.0–29.9), and obese (≥ 30.0).

*Laboratory analyses.* PFOS, PFOA, and PFHxS were measured in 448 stored maternal serum samples collected during 1991–1992 at pregnancy. The median gestational age when serum samples were obtained was 15 weeks, and the interquartile range was 10–28 weeks. Valid results were available for 447 girls. Serum samples were analyzed at the National Center for Environmental Health of the CDC (Atlanta, GA). Analytical methods used have been described elsewhere (Kuklenyik et al. 2005). Limits of detection were 0.2 ng/mL for PFOS and 0.1 ng/mL for PFOA and PFHxS. We prepared low-concentration (~ 2–9 ng/mL) and high-concentration (~ 6–25 ng/mL) quality control materials with pooled human serum that was analyzed with standards, reagent blanks, and study samples. Depending on the analyte, the precision of measurements, expressed as the relative SD, was 8–13%.

*Statistical analyses.* Our analyses were conducted on a sample of girls and their mothers previously selected for a nested case–control study to test associations of PFCs with earlier age at menarche. Because ignoring the sampling scheme can lead to biased results, we accounted for sampling selection probabilities by constructing stratum-weighted linear and longitudinal regression models to estimate the associations between PFC concentrations and fetal and postnatal growth (Richardson et al. 2007). The weight for the cases (all girls who attained menarche < 11.5 years) was 1, and the weight for the controls (a random sample of girls who attained menarche ≥ 11.5 years of age) was 15.1.

The markers of fetal growth studied were birth weight, birth length, gestational age, and ponderal index. We constructed stratum-weighted adjusted linear regression models to explore associations of each birth outcome with maternal serum concentrations of PFOA, PFOS, and PFHxS. We used the same linear regression methods to model outcomes at 20 months. The single-pollutant stratum-weighted models for each study outcome were constructed using backward elimination to identify potential confounders, with *p* < 0.20 as the cutoff for retention. Trend tests for analyses of associations with outcomes at birth and at 20 months were done where the exposure was modeled as an ordinal variable and coded as 0, 1, and 2. We tested whether exposure–response trends were significantly different from the null using a *p* < 0.05.

We examined the influence of missing values for covariates missing values for ≥ 10% of girls. The covariates missing values in this range were maternal smoking during pregnancy and maternal prepregnancy BMI. Comparisons of bivariate associations between the exposures and study outcomes before and after exclusion of girls missing data on either maternal prepregnancy BMI or smoking during pregnancy were conducted to verify for consistency. Results of such analyses suggested that exclusion of subjects missing values for theses covariates had the potential to introduce bias because changes in the magnitude and direction of bivariate associations were observed after these exclusions. Because maternal smoking and maternal pre-pregnancy BMI are predictors of the daughter’s growth, categories for missing values were included in the analyses of fetal and postnatal growth.

Linear mixed models were used for the longitudinal analyses of weight (Verbeke and Molenberghs 1997). We included girls who had 3 or 4 weight-for-age SD scores using weights obtained at birth or 2, 9, or 20 months (*n* = 410). The “time” variable represents the month of age when weight measurements were taken. These models were also constructed with sampling weights to account for the nested case–control sampling.

First, we created exploratory graphs to observe weight-for-age patterns over time by exposure and potential predictors (maternal prepregnancy BMI, maternal age at delivery, maternal educational level, previous live births, maternal smoking during pregnancy, gestational age, gestational age when maternal serum sample was obtained, and breast-feeding in the first 4 weeks). Weight-for-age patterns between 2 and 20 months appeared to be quadratic rather than linear. As described previously, a category for missing data was included for maternal smoking during pregnancy and maternal prepregnancy BMI. Then we fit saturated linear mixed-effects models for each of the three PFC exposures (exposures categorized as tertiles), and included intercept, age, and age-squared parameters for the exposures and potential predictors. To assess whether exposures or predictors influenced weight patterns, we included interactions between age and each covariate and age-squared and each covariate. We used the saturated model to test for random-effects and various random-effects covariance structures. It was appropriate to include a random intercept in addition to random slopes (for age and age-squared), with an unstructured random-effects covariance structure. We removed covariates in a hierarchical way, using backward elimination, to obtain a reduced model. The exposure was retained regardless of significance. Nested models were compared with likelihood ratio tests and potential predictors that were significant at the *p* < 0.20 were retained. Finally, we constructed graphs to depict the predicted mean weight-for-age SD scores, based on the final linear mixed-effects models, which were calculated at the 4 time points (birth and 2, 9 and 20 months), with PFC exposure as tertiles and all other covariates at their reference. All analyses were conducted using SAS version 9.2 (SAS Institute Inc., Cary, NC). Analyses of markers of fetal growth were performed in PROC GLM and the longitudinal mixed models were performed in PROC MIXED.

Human subject protection was assessed and approved by the ALSPAC Law and Ethics Committee, the local research ethics committees, and the CDC Institutional Review Board.

Informed consent was provided by the mothers at the time of enrollment. The daughters did not provide consent as such because they were children.

## Results

Analytes were detected in 100% of samples. PFOS showed the highest median maternal serum concentrations followed by PFOA, and PFHxS respectively (Table 1). Spearman correlation coefficients showed a high level of correlation between PFOS and PFOA (*r* = 0.72) and moderate to high levels between PFHxS and PFOS (*r* = 0.54) and PFHxS and PFOA (*r* = 0.44).

**Table 1:** Frequency distribution and maternal serum concentrations (in ng/mL) in 1991–1992 for selected study variables in a sample of British girls (*n* = 447).

Frequency [n (%)]		PFOS Median (min–max)	PFOA Median (min–max)	PFHxS Median (min–max)
Overall	447	(99.7)	19.6 (3.8–112.0)	3.7 (1.0–16.4)	1.6 (0.2–54.8)
Maternal prepregnancy BMI
Underweight	18	(4.03)	16.9 (11.1–42.9)	3.5 (1.2–8.3)	1.5 (0.7–3.6)
Normal	290	(64.88)	20.1 (3.8–112.0)	3.8 (1.0–15.7)	1.6 (0.2–54.1)
Overweight	62	(13.87)	20.9 (11.3–47.6)	3.7 (1.9–6.5)	1.9 (0.7–17.5)
Obese	31	(6.94)	19.2 (9.4–74.2)	3.6 (2.1–11.1)	1.4 (0.9–4.4)
Missinga	46	(10.29)	17.3 (8.5–94.5)	3.5 (1.3–16.4)	1.6 (0.5–54.8)
Maternal age at delivery (years)
< 25	92	(20.58)	18.5 (9.2–32.6)	3.9 (1.8–8.6)	1.6 (0.8–6.2)
25–29	164	(36.69)	20.7 (6.5–112)	3.8 (1.2–16.4)	1.6 (0.6–54.8)
≥ 30	188	(42.06)	19.6 (3.8–74.2)	3.6 (1.0–15.7)	1.7 (0.2–49.8)
Missing	3	(0.67)
Maternal education
< O level	89	(19.91)	8.2 (8.94.5)	3.6 (1.3–16.4)	6 (0.4–54.8)
O level	140	(31.32)	19.6 (8.5–112.0)	3.7 (1.6–8.6)	1.6 (0.5–37.3)
> O level	199	(44.52)	20.4 (3.8–69.2)	3.9 (1.0–15.7)	1.7 (0.2–54.1)
Missing	19	(4.25)
Maternal race
White	422	(94.41)	19.9 (6.5–112.0)	3.8 (1.1–16.4)	1.6 (0.2–54.8)
Nonwhite	8	(1.79)	14.6 (3.8–25.6)	2.3 (1.0–3.5)	1.4 (0.3–2.8)
Missing	17	(3.80)
Previous live births
0	212	(47.43)	21.5 (6.5–94.5)	4.4 (1.5–16.4)	1.8 (0.4–54.8)
≥ 1	215	(48.10)	18.2 (3.8–74.2)	3.1 (1.0–13.8)	1.5 (0.2–49.8)
Missing	20	(4.47)
Smoking during pregnancy
Yes	85	(19.02)	17.2 (7.6–39.6)	3.4 (1.2–7.5)	1.6 (0.2–9.5)
No	318	(71.14)	20.9 (4.3–112.0)	3.9 (1.0–16.4)	1.7 (0.3–54.8)
Missinga	44	(9.84)	18.0 (3.8–74.2)	3.2 (1.1–11.1)	1.4 (0.4–24.9)
Low birth weight
Yes	17	(3.80)	21.8 (4.3–112.0)	4.1 (1.0–11.1)	1.7 (0.3–37.3)
No	422	(94.41)	19.6 (3.8–94.5)	3.7 (1.1–16.4)	1.6 (0.2–54.8)
Missing	8	(1.79)
Preterm delivery
Yes	14	(3.13)	23.9 (10.9–112.0)	4.7 (2.5–8.5)	1.7 (0.9–37.3)
No	430	(96.20)	19.6 (3.8–94.5)	3.7 (1.0–16.4)	1.6 (0.2–54.8)
Missing	3	(0.67)
Breast-feeding in first 4 weeks
Yes	358	(80.09)	19.9 (3.8–112.0)	3.7 (1.0–16.4)	1.6 (0.2–54.8)
No	74	(16.55)	18.2 (8.5–39.6)	3.7 (2.1–6.7)	1.7 (0.6–7.1)
Missing	15	(3.36)
Menarche (years)
< 11.5	218	(48.77)	19.5 (3.8–112.0)	3.9 (1.0–15.7)	1.7 (0.3–7.3)
> 11.5	229	(51.23)	19.9 (7.6–94.5)	3.6 (1.1–16.4)	1.6 (0.2–54.8)
Abbreviations: max, maximum; min, minimum. aCategory for missing values was included in the analyses.									

Among the 447 girls mean birth weight (± SD) was 3,397 ± 449 g; birth length was 50.4 ± 2.2 cm; ponderal index was 2.7 ± 0.2 kg/m^3^; and gestational age was 39.8 ± 1.6 weeks. Fewer than 4% of girls were preterm or born weighing < 2,500 g. About half of the mothers reported a normal maternal prepregnancy BMI and having had at least one previous live birth. Most mothers were white (95%) (Table 1).

Sample sizes for multivariate analyses varied from 320 to 395 in the main analyses and from 106 to 107 in stratified analyses. On average, girls born to mothers with maternal serum concentrations of PFOS in the upper tertile weighed 140 g [95% confidence interval (CI): –238, –42] less at birth compared with girls born to mothers with serum concentrations in the lower tertile in models adjusted for gestational age, maternal prepregnancy BMI, previous live births, and maternal smoking during pregnancy (Table 2). Girls born to mothers with maternal serum concentrations of PFOS in the middle tertile also weighed less at birth, on average, than did girls born to mothers with serum concentrations in the lower tertile. Similar magnitudes of weight differences were observed between girls whose mothers had the highest and lowest serum concentrations of PFOA (–133 g; 95% CI: –237, 30) and PFHxS (–108 g; 95% CI: –206, –10). Results for associations of birth length with PFOS and PFHxS showed similar patterns to those observed for birth weight in models adjusted for gestational age, maternal educational level, maternal prepregnancy BMI, previous live births, and maternal smoking during pregnancy. Trend tests suggest exposure–response gradients across tertiles of maternal serum concentrations of the three PFCs tested for birth weight and across tertiles of PFOS and PFHxS but not PFOA for birth length. Results for gestational age and ponderal index did not suggest an association with the PFCs tested (Table 2).

**Table 2:** Adjusted least square means (LSM) and regression coefficients (β) for prenatal growth measures by tertiles of PFOS, PFOA, and PFHxS in a sample of British girls in 1991–1992.

Tertiles of analyte (ng/mL)	Birth weighta (g) (n = 422)				Birth lengthb (cm) (n = 356)				Gestational agec (weeks) (n = 444)				Ponderal indexd (n = 360)			
	LSM	β (95% CI)		LSM	β (95% CI)		LSM	β (95% CI)		LSM	β (95% CI)	
PFOS
< 16.6	3438.59	Reference		50.91	Reference		39.84	Reference		2.66	Reference	
16.6–23.0	3326.88	–111.71	(–208.24, –15.17)	50.19	–0.72	(–1.19, –0.25)	39.82	–0.02	(–0.39, 0.35)	2.66	–0.00	(–0.07, 0.06)
> 23.0	3298.57	–140.01	(–238.14, –41.89)	50.28	–0.63	(–1.11, –0.15)	39.69	–0.15	(–0.53, 0.23)	2.71	0.05	(–0.01, 0.12)
Trend test p-value			0.0053		0.0103		0.4352		0.1120	
PFOA
< 3.1	3419.22	Reference		50.54	Reference		39.96	Reference		2.69	Reference	
3.1–4.4	3362.41	–56.81	(–153.05, 39.43)	50.67	0.14	(–0.34, 0.61)	39.72	–0.25	(–0.61, 0.12)	2.63	–0.06	(–0.12, 0.01)
> 4.4	3285.77	–133.45	(–237.37, –29.54)	50.10	–0.44	(–0.96, 0.08)	39.62	–0.34	(–0.73, 0.05)	2.71	0.02	(–0.05, 0.09)
Trend test p-value			0.0120		0.0978		0.0833		0.5920	
PFHxS
< 1.3	3402.21	Reference		50.93	Reference		39.91	Reference		2.67	Reference	
1.3–2.0	3393.10	–9.10	(–108.08, 89.88)	50.41	–0.52	(–1.00, –0.04)	39.76	–0.15	(–0.52, 0.22)	2.70	0.03	(–0.04, 0.09)
> 2.0	3294.27	–107.93	(–206.18, –9.69)	50.11	–0.82	(–1.29, –0.34)	39.67	–0.24	(–0.62, 0.14)	2.65	–0.01	(–0.08, 0.05)
Trend test p-value			0.0314		0.0008		0.2170		0.6802	
For maternal prepregnancy BMI and smoking during pregnancy, categories for missing values were included in the analyses. aAdjusted by maternal smoking during pregnancy, maternal prepregnancy BMI, previous live births, and gestational age. bAdjusted by maternal smoking during pregnancy, maternal prepregnancy BMI, maternal education, previous live births, and gestational age. cAdjusted by gestational age when maternal serum sample was obtained. dAdjusted by maternal prepregnancy BMI, previous live births, and gestational age when maternal serum sample was obtained.																				

Pearson correlation coefficients showed strong correlations between birth weight and weight at 20 months (*r* = 0.32; *p* < 0.0001; *n* = 339), birth weight and height at 20 months (*r* = 0.20; *p* < 0.0003; *n* = 339), and weight at 20 months and height at 20 months (*r* = 0.67; *p* < 0.0001; *n* = 343). In adjusted analyses of weight at 20 months, when either birth weight or height at 20 months or both were included in the model, girls with the highest maternal serum concentrations were on average between 438 and 580 g heavier than girls with maternal serum concentrations in the lower tertile (Table 3). Weight at 20 months was not associated with the other PFCs.

**Table 3:** Adjusted least square means (LSM) and regression coefficients (β) for weight (g) at 20 months by tertiles of PFOS, PFOA, and PFHxS in a sample of British girls in 1991–1992.

Tertiles of analyte (ng/mL)	Weight at 20 monthsa (g) (n = 324)				Weight at 20 months (g) adjusted by birth weighta (n = 320)				Weight at 20 months (g) adjusted by height at 20 monthsa (n = 324)				Weight at 20 months (g) adjusted by height at 20 months and birth weighta (n = 320)			
	LSM	β (95% CI)		LSM	β (95% CI)		LSM	β (95% CI)		LSM	β (95% CI)	
PFOS
< 16.6	11412.6	Reference		11418.9	Reference		11363.9	Reference		11360.1	Reference	
16.6–23.0	11513.6	100.93	(–282.81, 484.68)	11529.4	110.4	(–262.70, 483.60)	11678.5	314.60	(19.37, 609.82)	11670.7	310.64	(27.19, 594.08)
> 23.0	11777.0	364.35	(–15.14, 743.83)	11857.4	438.4	(71.09, 805.65)	11863.7	499.84	(208.67, 791.01)	11939.9	579.82	(301.40, 858.25)
Trend test p-value			0.0598		0.0195		0.0008		< 0.0001	
PFOA
< 3.1	11635.0	Reference		11597.0	Reference		11737.2	Reference		11695.3	Reference	
3.1–4.4	11460.7	–174.31	(–550.55, 201.94)	11512.3	–84.75	(–452.58, 283.08)	11477.0	–260.24	(–550.48, 30.00)	11511.1	–184.21	(–465.90, 97.48)
> 4.4	11644.5	9.48	(–405.48, 424.44)	11739.3	142.31	(–262.06, 546.67)	11752.1	14.93	(–304.92, 334.783)	11823.7	128.40	(–180.94, 437.74)
Trend test p-value			0.9642		0.4892		0.9269		0.4147	
PFHxS
< 1.3	11566.2	Reference		11531.2	Reference		11664.8	Reference		11624.9	Reference	
1.3–2.0	11534.4	–31.84	(–416.87, 353.20)	11558.8	27.58	(–346.32, 401.49)	11632.0	–32.83	(–331.30, 265.65)	11637.3	12.33	(–275.56, 300.22)
> 2.0	11629.1	62.86	(–326.68, 452.40)	11739.6	208.43	(–170.60, 587.46)	11652.3	–12.55	(–314.69, 289.58)	11740.4	115.40	(–176.69, 407.50)
Trend test p-value			0.7511		0.2801		0.9349		0.4375	
aValues were also adjusted by maternal education, maternal age at delivery, and previous live birth.																				

To better understand the role of birth weight in the association between weight at 20 months and exposure to PFOS, we further stratified weight at 20 months by birth weight and tertiles of exposure (Table 4). Among girls in the upper birth weight stratum, the highest serum maternal concentrations of PFOS were on average 600 g (95% CI: –53, 1,245) heavier at 20 months than those with the lowest serum concentrations. Among girls in the middle birth weight stratum, we did not find weight at 20 month to be associated with PFOS. Finally, among girls in the lowest birth weight stratum those with the highest maternal serum concentrations of PFOS where on average 933 g (95% CI: 187, 1,679) heavier than those with the lowest serum concentrations.

**Table 4:** Adjusted least square means (LSM) and regression coefficients (β) for weight at 20 months stratified by tertiles of PFOS, PFOA, and PFHxS and tertiles of birth weight^a^ in a sample of British girls in 1991–1992.

	Upper tertile > 3,580 g (n = 106)				Middle tertile 3,200–3,580 g (n = 107)				Lower tertile < 3,200 g (n = 107)			
	LSM	β (95% CI)		LSM	β (95% CI)		LSM	β (95% CI)	
PFOS
< 16.6	11577.29	Reference		11659.86	Reference		10827.14	Reference	
16.6–23.0	11910.86	333.57	(–301.28, 968.42)	11397.04	–262.83	(–884.25, 358.60)	11429.78	602.64	(–150.79, 1356.07)
> 23.0	12173.51	596.22	(–52.98, 1245.42)	11825.29	165.43	(–439.52, 770.37)	11759.85	932.71	(186.90, 1678.52)
Trend test p-value			0.0714		0.5886		0.0148	
PFOA
< 3.1	11906.83	Reference		11598.90	Reference		11259.57	Reference	
3.1–4.4	11921.96	15.13	(–573.62, 603.87)	11477.35	–121.55	(–708.11, 465.01)	11238.43	–21.13	(–827.99, 785.72)
> 4.4	11879.43	–27.39	(–785.40, 730.61)	11768.73	169.83	(–497.87, 837.54)	11507.84	248.27	(–570.54, 1067.08)
Trend test p-value			0.9430		0.6149		0.5488	
PFHxS
< 1.3	11854.12	Reference		11738.45	Reference		11003.43	Reference	
1.3–2.0	11782.43	–71.69	(–668.06, 524.68)	11511.75	–226.70	(–847.25, 393.84)	11326.72	323.28	(–453.95, 1100.51)
> 2.0	12159.19	305.07	(–404.41, 1014.54)	11687.39	–51.07	(–666.53, 564.40)	11601.69	598.26	(–166.91, 1363.43)
Trend test p-value		0.3955		0.8696		0.1240	
aAdjusted by maternal education, maternal age at delivery, previous live birth, and birth weight as a continuous variable.															

**Figure 1:**
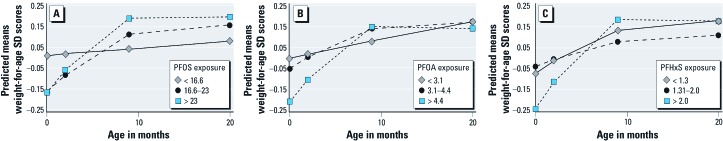
Predicted means for weight-for-age SD scores by age in months and tertiles of maternal serum concentrations of PFOS (*A*), PFOA (*B*), and PFHxS (*C*) from mixed models, adjusted by previous live birth and maternal smoking during pregnancy. For maternal smoking during pregnancy, a category for missing ­values was included in the analyses.

Figure 1 gives the predicted mean weight-for-age SD scores by PFC exposure tertiles (predicted at birth and 2, 9, and 20 months) based on the longitudinal mixed models. The corresponding coefficients from the mixed models are provided in Supplemental Material, Table S1 (http://dx.doi.org/10.1289/ehp.1003096). The initial points on the figures represent the intercept coefficients for weight at birth. The estimates for the intercept coefficients were consistent with the analyses of PFC exposures with birth weight: Girls in the upper tertile of exposure weighed less than the girls in the lower tertile. Girls in the middle tertile weighed less than girls in the lower tertile. However, the *p*-values for the differences between the intercept coefficients did not reach statistical significance (PFOS, *p* = 0.28; PFOA, *p* = 0.27; PFHxS, *p* = 0.21).

The remaining points on Figure 1 represent predicted mean weight-for-age SD scores by PFC exposure for 2, 9, and 20 months. For girls in the lowest tertile of exposure to PFOS, there is very little change in weight-for-age SD score over time (they remain around the median weight-for-age at each time point). However, the trajectory for girls in the middle and upper exposure tertiles surpass the lower tertile group by 9 months. Further, the girls in the middle- and upper-tertile groups remain heavier at 20 months than the girls in the lower tertile group. For the PFOA and the PFHxS figures, the trajectory of weight-for-age SD scores follow a more similar pattern for the girls in the lower and middle exposure tertile groups, whereas by 9 months the girls in the upper tertile groups exceed (for PFOA) or reach (PFHxS) the weight of the girls in the lower and middle tertile groups. Although the figure suggests that the weight-for-age trajectories appear to vary to some extent by exposure tertiles over time, interaction term coefficients for time and tertiles of exposure from the mixed models were not statistically significant (*p* > 0.05) [see Supplemental Material, Table S1 (http://dx.doi.org/10.1289/ehp.1003096)].

As in the single time point analyses of weight at 20 months, we were interested in understanding the role of birth weight in the weight-for-age SD patterns between 2 and 20 months. To do so, we removed birth weight as a time point in the longitudinal analyses and reran the mixed models with three time points (2, 9, and 20 months), and stratified by birth weight (data not shown). In these models, for the intercept we found that within the lower birth weight strata, girls were smaller at birth in the higher-exposure tertile than those in the lower-exposure tertile. This was also seen for the upper birth weight strata. The coefficients for girls in the middle birth weight group were larger with higher PFC concentrations. The time trend coefficients followed a similar pattern for the girls in the lower and upper birth weight groups, compared with the middle birth weight group.

## Discussion

Evidence from animal and human studies suggests that prenatal exposures to PFCs may have deleterious effects on fetal and postnatal growth. We explored associations of maternal serum concentrations of PFOS, PFOA, and PFHxS during pregnancy with markers of fetal and postnatal growth in a sample of British girls. In our study we found that girls born to mothers with higher serum PFC concentrations during pregnancy were smaller at birth. The magnitude of the differences in birth weight and length by tertile was similar across all the PFCs evaluated, and an exposure–response gradient was observed. Girls with higher prenatal concentrations of the PFC also appear to be heavier at 20 months.

The PFCs most commonly assessed in other epidemiologic studies of fetal growth were PFOA and PFOS. Our results are consistent with those reported by others, although the magnitude of the differences in birth weight and length are not strictly comparable between studies because of differences in analytical strategy or biological media where PFCs were assessed (Apelberg et al. 2007; Arbuckle et al. 2012; Washino et al. 2009). Two studies looked at PFHxS and birth weight (Arbuckle et al. 2012; Hamm et al. 2010). Although both found no association, exposure levels were quite low, with a large proportion of samples without detectable PFHxS. Maternal serum concentrations of PFOS, PFOA, and PFHxS in our study were higher than those reported in other population-based studies of fetal growth conducted in Japan (Washino et al. 2009), Canada (Hamm et al. 2010), and the United States (Apelberg et al. 2007) but lower than those reported in a Danish study (Fei et al. 2008, 2007). Except for the Danish study, where serum sample collection began in 1996, sample collection in other studies began after the year 2000. Maternal serum samples in our study were collected in the early 1990s, when worldwide production rates of PFOS, PFOA, and presumably PFHxS were greater than current rates. The higher maternal concentrations of PFCs in our study may explain, in part, the consistency of our associations across the PFCs evaluated. The observed shift in birth weight distribution in association with *in utero* PFC exposure is smaller than those reported for maternal smoking. However, because PFCs are ubiquitous in the environment, human exposure is common and, for the most part, involuntary, altered prenatal growth due to PFC exposure may represent an important public health issue.

The greatest variations in rates of weight gain are usually seen in the first 2 years of life, when infants show accelerated or diminished growth to compensate for intrauterine restraint or enhancement of fetal growth (Ong et al. 2000). In our analyses, we found that girls with higher prenatal exposures to the PFCs examined were smaller at birth and heavier at 20 months. A recently published study found that *in utero* exposure to PFOA, but not PFOS, was positively associated with BMI and weight circumference at 20 years in females, suggesting that obesity could be associated with gestational PFC exposures (Halldorsson et al. 2012).

Relationships between the PFCs and patterns in weight-for-age SD scores between birth and 20 months described by plotting predicted means from longitudinal models were not linear. Adiposity increases in the first year of life and subsequently decreases (Rolland-Cachera et al. 1987). We have previously reported an adiposity decrease after the first year of life in ALSPAC girls (Maisonet et al. 2010). This decrease suggests a slower pace of weight gain between 9 and 20 months compared with the period between birth and 9 months in ALSPAC girls. In our study, age points when postnatal weight and height data were obtained were not established *a priori* but determined by the available data.

Our analysis was conducted on a sample of girls and their mothers selected for a nested case–control study of pubertal development. For this reason we used weighted regression models to estimate the associations between PFC maternal serum concentrations and fetal and postnatal growth to adjust for the sampling scheme. Our results could be biased if girls with missing data (excluded from analysis) were different from girls who were included in these analyses. Mean values of the study outcomes and maternal characteristics for girls included in final multivariate analyses were similar to the group of girls enrolled in the cohort (~ 3,000), suggesting that selection bias is an unlikely explanation for our results. Finally, although we were unable to examine postnatal exposure to PFCs and impact on postnatal growth in this study, this is an important question for future research.

## Conclusion

Girls born to mothers with higher serum concentrations of PFOS, PFOA, and PFHxS during pregnancy appear to be smaller at birth and heavier at 20 months.

## Supplemental Material

(82 KB) PDF
