# Supplementary material for: Maternal Concentrations of Polyfluoroalkyl Compounds during Pregnancy and Fetal and Postnatal Growth in British Girls
Source: Environ Health Perspect. 2012 Aug 30;120(10):1432–7. doi: 10.1289/ehp.1003096 (PMC3491920; doi:10.1289/ehp.1003096)
Supplement: (82 KB) PDF [file ehp.1003096.s001.pdf]

Supplemental Material

Maternal Concentrations of Polyfluoroalkyl Compounds during Pregnancy and Fetal and Postnatal Growth in British Girls

M Maisonet, ML Terrell, MA McGeehin, KY Christensen, A Holmes, AM Calafat, M Marcus

Table S1. Parameter estimates obtained from fitting linear mixed-effects models for weight-for-age SD scores at birth, 2, 9, and 20 months in British girls, including serum concentrations of PFOS, PFOA or PFHxS during pregnancy (n=395)

Variables	PFOS		PFOA		PFHxS	
	B	p-value	β	p-value	β	p-value
PFC (lower tertile) [a]	0.011		-0.004		-0.073	
PFC (middle tertile) [a]	-0.164	0.28	-0.053	0.27	-0.041	0.21
PFC (upper tertile) [a]	-0.166		-0.212		-0.244	
Previous live births (1+)	0.401	<0.01	0.363	<0.01	0.414	<0.01
Smoking during pregnancy (yes)[b]	-0.292	0.09	-0.283	0.10	-0.267	0.13
Smoking during pregnancy (missing)[b]	-0.130		-0.137		-0.104	
Age∗PFC (lower tertile) [a]	0.003		0.010		0.031	
Age∗PFC (middle tertile) [a]	0.043	0.11	0.029	0.23	0.018	0.11
Age∗PFC (upper tertile) [a]	0.057		0.058		0.070	
Age∗Previous live births (1+)	-0.060	0.01	-0.053	0.02	-0.066	<0.01
Age∗Smoking during pregnancy (yes)[b]	0.088	0.01	0.084	0.01	0.079	0.02
Age∗Smoking during pregnancy (missing)[b]	0.001		0.001		-0.009	
Age^2∗PFC (lower tertile) [a]	0.000002		-0.00004		-0.00094	
Age^2∗PFC (middle tertile) [a]	-0.00134	0.19	-0.00091	0.24	-0.00051	0.15
Age^2∗PFC (upper tertile) [a]	-0.00195		-0.00201		-0.00243	
Age^2∗Previous live births (1+)	0.00237	0.01	0.00205	0.03	0.00259	<0.01
Age^2∗Smoking during pregnancy (yes)[b]	-0.00327	0.02	-0.00313	0.02	-0.00291	0.03
Age^2∗Smoking during pregnancy (missing)[b]	0.00015		0.00018		0.00056	

[a] PFC tertiles: PFOS: <16.6, 16.6-23.0, >23.0; PFOA: <3.1, 3.1-4.4, >4.4; PFHxS: <1.3, 1.3-2.0, >2.0
Chunk Tests: H_o: PFC= Age∗PFC= Age^2∗PFC=0; PFOS, p=0.81; PFOA, p=0.89; PFHxS, p=0.79
[b] For maternal smoking during pregnancy a category for missing values was included in the analyses.
